# 3D Printing in Surgical Management of Double Outlet Right Ventricle

**DOI:** 10.3389/fped.2017.00289

**Published:** 2018-01-10

**Authors:** Shi-Joon Yoo, Glen S. van Arsdell

**Affiliations:** ^1^Department of Diagnostic Imaging, Hospital for Sick Children, University of Toronto, Toronto, ON, Canada; ^2^Division of Cardiology, Department of Paediatrics, Hospital for Sick Children, University of Toronto, Toronto, ON, Canada; ^3^Division of Cardiovascular Surgery, Department of Surgery, Hospital for Sick Children, University of Toronto, Toronto, ON, Canada

**Keywords:** double outlet right ventricle, congenital heart surgery, 3D printing, surgical simulation, hands-on surgical training

## Abstract

Double outlet right ventricle (DORV) is a heterogeneous group of congenital heart diseases that require individualized surgical approach based on precise understanding of the complex cardiovascular anatomy. Physical 3-dimensional (3D) print models not only allow fast and unequivocal perception of the complex anatomy but also eliminate misunderstanding or miscommunication among imagers and surgeons. Except for those cases showing well-recognized classic surgical anatomy of DORV such as in cases with a typical subaortic or subpulmonary ventricular septal defect, 3D print models are of enormous value in surgical decision and planning. Furthermore, 3D print models can also be used for rehearsal of the intended procedure before the actual surgery on the patient so that the outcome of the procedure is precisely predicted and the procedure can be optimally tailored for the patient’s specific anatomy. 3D print models are invaluable resource for hands-on surgical training of congenital heart surgeons.

## Introduction

Double outlet right ventricle (DORV) has long been a contentious topic in congenital heart pathology and surgery (1). DORV is defined as a type of ventriculoarterial connection in which both great arteries arise either entirely or predominantly from the right ventricle (1, 2). DORV shows extremely variable morphologic features and clinical presentations (1, 2). Therefore, surgical management of the patients with DORV should be individualized according to the given morphologic and hemodynamic status. Although biventricular repair is ideal, it is often not applicable. Inappropriate decision toward biventricular repair can be catastrophic, while inadvertent decision for univentricular repair leaves the patients with complications of high systemic venous pressures and low cardiac output for life.

Traditionally, surgical decision and planning relied on echocardiography (2). Recently, there has been increasing utilization of computed tomography (CT) and magnetic resonance (MR) for clear demonstration of the surgical anatomy as both imaging modalities provide 3-dimensional (3D) volume data of the cardiovascular system with relatively high spatial and temporal resolutions. As CT and MR imaging is not hampered by the presence of air or bones and does not require any defined access windows for imaging as in echocardiogrpahy, 3D-volume data are readily applicable for 3D demonstration of the structures of interest on a computer screen, which greatly facilitates appreciation and understanding of complex geometry of congenital heart diseases. However, virtual demonstration can be liable to misrepresentation and misinterpretation as demonstration is on a two-dimensional computer screen. 3D printing fills the gap between the virtual and physical reality (3–5). With the physical models on the observer’s hands, the chances of misinterpretation and miscommunication at surgical planning can almost completely be eliminated. Furthermore, 3D print models allow physical as well as mental simulation of planned surgical procedure (6).

In this review article, we review the utility and future of 3D reconstruction and printing in surgical management of the patients with DORV based on our experience in the last 10 years and literature review (7–10).

## Imaging

3D printing requires 3D-volumetric imaging data. Contrast-enhanced CT or MR angiograms are best suited for 3D imaging and printing in DORV. Electrocardiographically (ECG) gated imaging with breath-holding or respiration navigation provides the best quality images and models. Non-ECG-gated imaging with spontaneous shallow breathing is not ideal but applicable with limited appreciation of fine structures. In general, CT provides the highest spatial resolution (<0.3 mm), while MR hardly provides less than a millimeter resolution because of a limited signal-to-noise ratio and prolonged scan time. MR may provide a higher temporal resolution than CT at the expense of scan time. ECG-gated and respiration navigated MR angiograms consistently provide homogeneous signal intensity throughout the blood pool, while various grades of inhomogeneous signal intensity among regions are hardly avoidable in CT. Nonetheless, a special attention should be paid to achieve the signal intensity of the blood pool as homogeneous as possible throughout the cardiac cavities and major vessels (11–13). Rotational X-ray angiograms are also applicable but are rarely performed (14). Echocardiography also provides 3D-volumetric image data for 3D printing but is of limited utility because of shadowing and artifact from bones and air. However, echocardiography is advantageous over CT and MR for visualization of the rapidly moving or thin structures such as atrial septum, tricuspid and mitral valve leaflets and their supporting structures (15–20). As none of the currently available imaging modalities is ideal for all cardiovascular structures, the data sets from multiple imaging modalities can be digitally coregistered to create a composite 3D model (19, 20).

## Postprocessing

**Figure 1:**
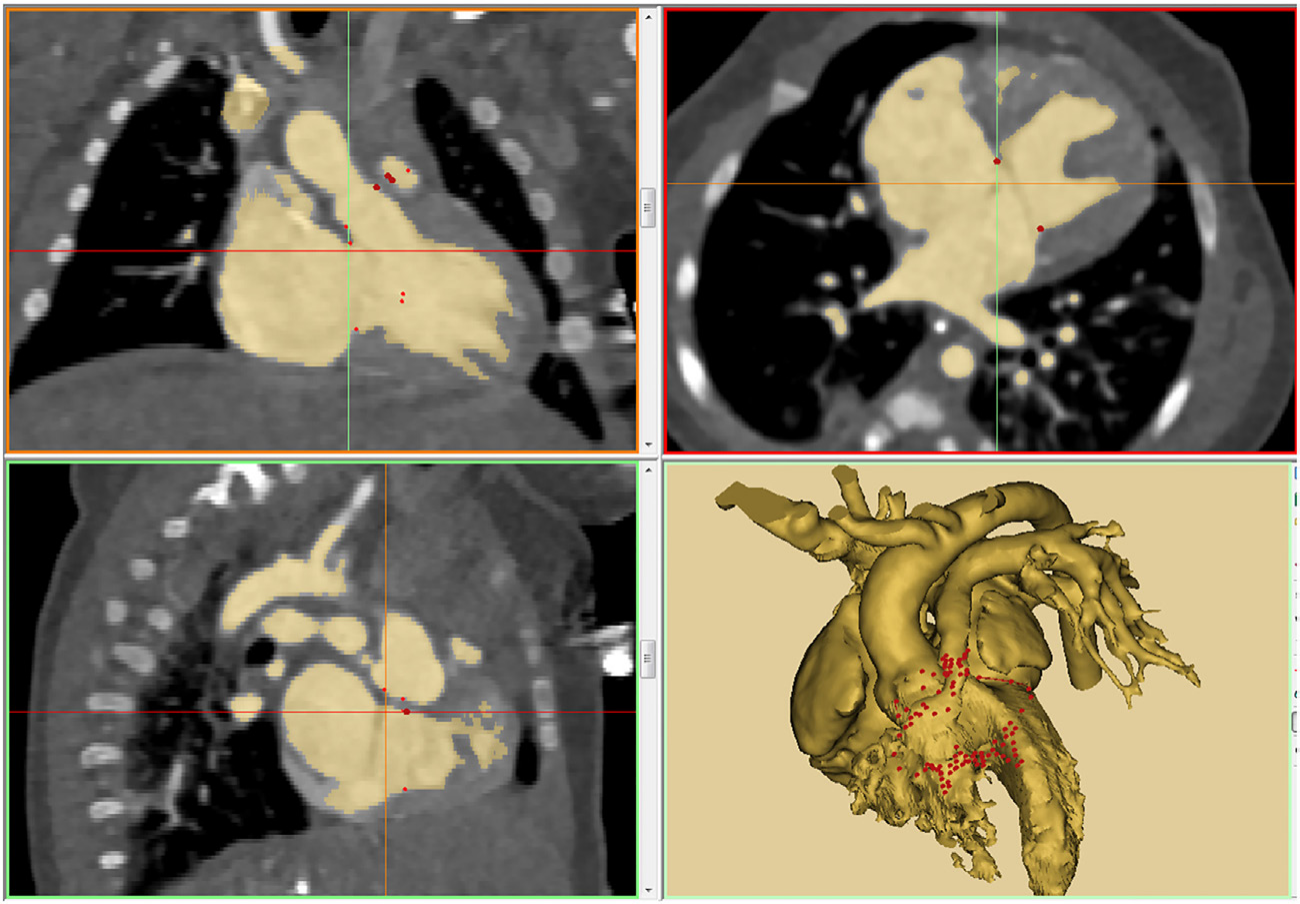
An example of thresholding on a patient with double outlet right ventricle (DORV) with atrioventricular septal defect and severe subpulmonary stenosis (tetralogy type of DORV with atrioventricular septal defect). The result of thresholding is displayed in three orthogonal planes of gray-scale images and 3D-volume rendered model. Note that the insertion sites of the cardiac valves are marked using “spline” function.

Postprocessing is the major determining factor of the quality of the products of 3D printing for the given image data. There are several software programs ranging from expensive high-end commercial versions to open-source freeware platforms that can be chosen according to the desired goals (21, 22). The original digital imaging and communication in medicine (DICOM) image data are required for postprocessing. Postprocessing consists of two steps: segmentation and computer-aided design (CAD). Segmentation implies identification of the regions of interest in the gray-scale images (Figure 1). The first step in segmentation is performed by using a uniform threshold range. The threshold-based segmented data are then manually edited using tools including “erasing,” “drawing,” and “local thresholding.” Manual editing is the most time consuming and laborious step in postprocessing. Segmentation requires understanding of the limitations and pitfalls of the imaging modalities as well as the pathologic anatomy of various congenital heart diseases. After the regions of interest are segmented, 3D-volume object is created. Currently available imaging modalities do not provide clear delineation of the valve structures for 3D printing. However, the sites of valvar attachments are definable on both 2D and 3D images (Figure 1, right lower panel). Six to ten valvar insertion points are marked on the 2D and/or 3D images and connected using the function called “spline.”

**Figure 2:**
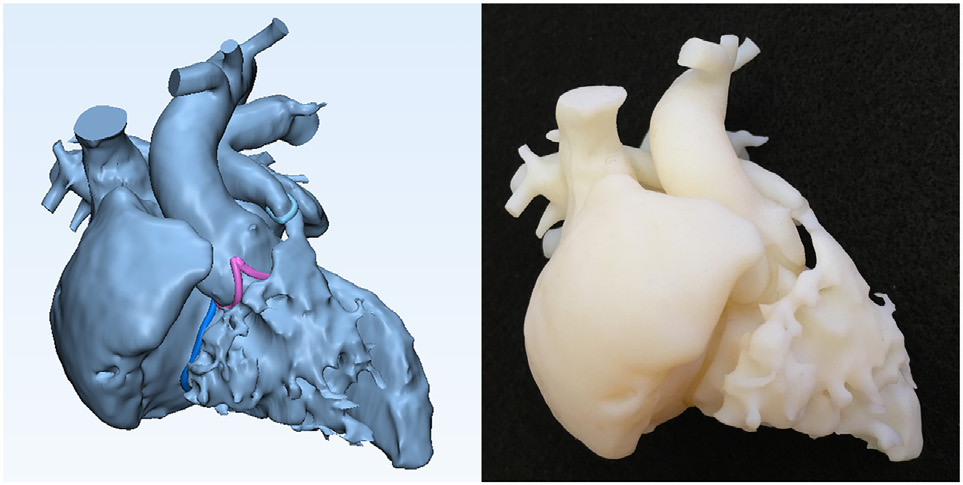
Graphic representation and photograph of the cast model made of solid material (Verowhite, Stratasys Ltd., MN, USA) of the case shown in Figure 1.

**Figure 3:**
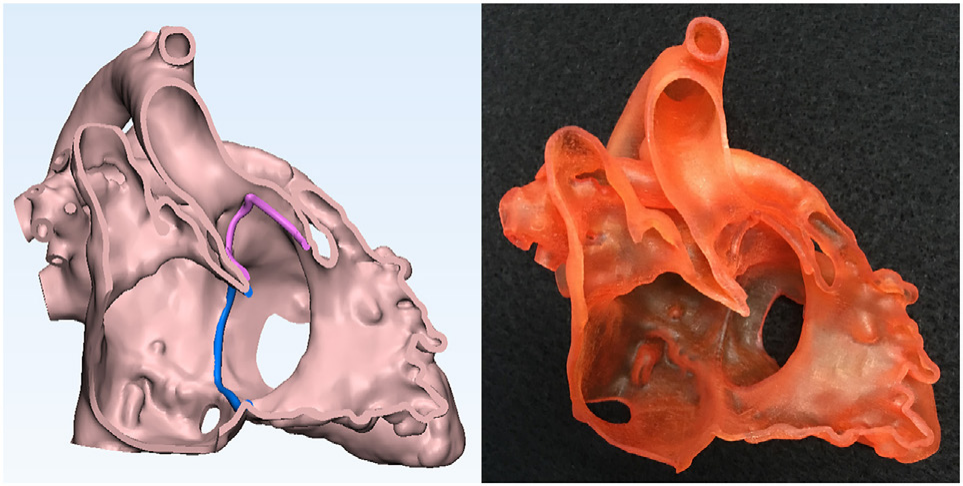
Graphic representation and photograph of the endocardial surface anatomy model made of soft material (TangoPlus, Stratasys Ltd., MN, USA) of the right ventricle of the case shown in Figure 1.

**Figure 4:**
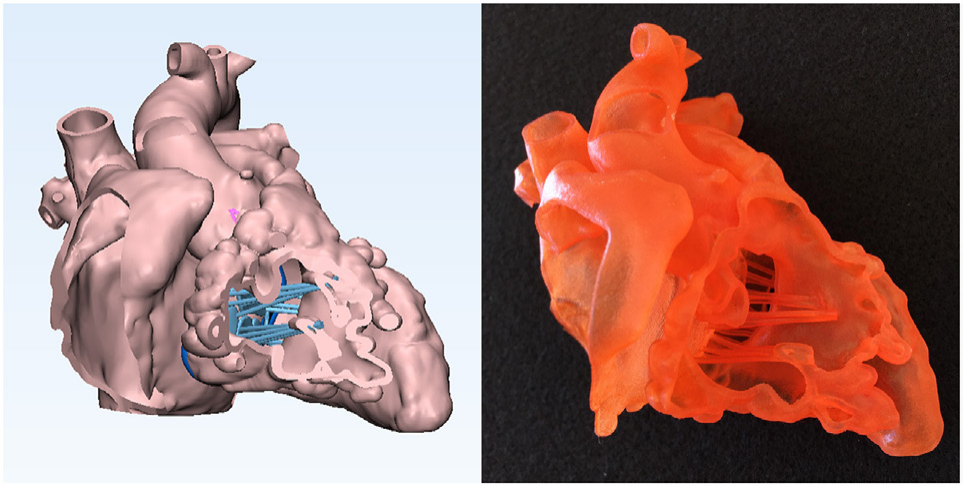
Graphic representation and photograph of the endocardial surface anatomy model made of soft material (TangoPlus, Stratasys Ltd., MN, USA) of the right ventricle of the case shown in Figure 1. The cardiac valve leaflets and the supporting structures of the common atrioventricular valve are graphically designed and added to the model shown in Figure 3.

**Figure 5:**
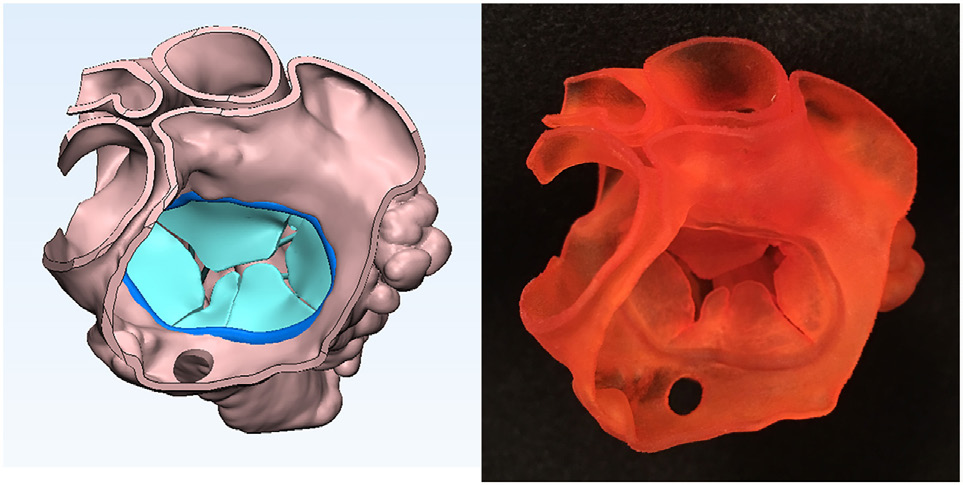
Graphic representation and photograph of the endocardial surface anatomy model made of soft material (TangoPlus, Stratasys Ltd., MN, USA) of the common atrium seen from behind and above of the case shown in Figure 1. Note the common atrioventricular valve leaflet orientation.

**Figure 6:**
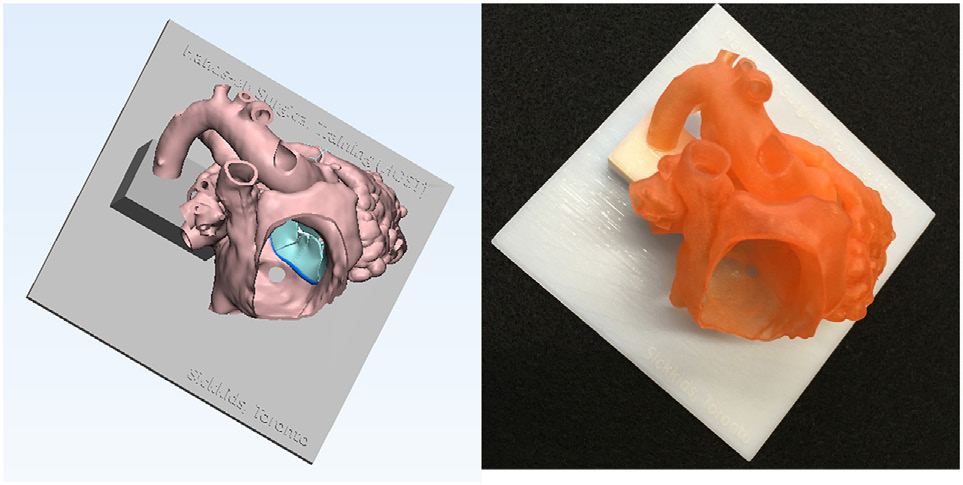
Graphic representation and photograph of the hands-on surgical training model. The endocardial surface anatomy model of the case shown in Figure 1 is designed in such a way that the surgical procedure can be simulated from the right atrium. To secure the position of the heart during the procedure, the model is mounted on a flat plate and supported by a column that are made of solid material (Verowhite, Stratasys Ltd., MN, USA).

**Figure 7:**
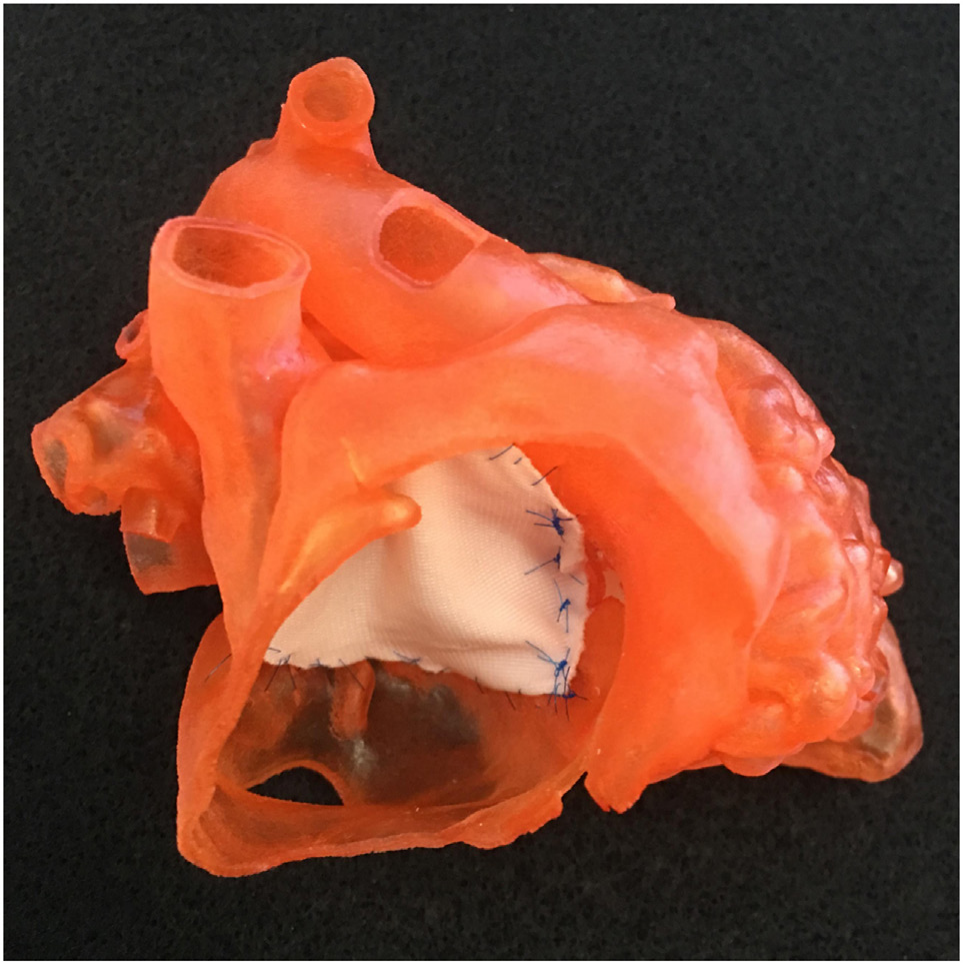
Photograph of a model shown in Figure 1 with a single patch applied to the atrioventricular septal defect.

The final 3D-volume rendered DICOM image files are converted to STL (Stereolithography or Standard Tessellation Language) or other applicable file format for CAD and 3D printing (Figures 2–7). We routinely reconstruct three models: a cast model of the blood pool, an endocardial surface anatomy model for septal views, and an endocardial surface anatomy model for basal view. Additional models are printed when required for better visualization of the complex anatomy. The cast model is a simple physical representation of the volume rendered angiogram and facilitates general understanding of the 3D anatomy in correlation with the images. The endocardial surface anatomy models are for appreciation of surgical anatomy at the operation table. Although cast models can be printed directly from the 3D-volume rendered angiograms, we empty the space inside and leave 1.2–1.5 mm of the surface using a CAD function “inside hollow” so that the inner empty space is filled with cheap support material (Figure 2). For representation of the surgical anatomy, a shell with an even thickness of 1–2 mm is created on the 3D-volume model using a CAD function “outside hollow.” In this outside-hollowed model, the inner surface represents the endocardial surface anatomy, whereas the outer surface is a false structure (Figure 3). Parts of the free wall of the atria and ventricles are removed in such ways to visualize the ventricular septal and outflow tract anatomy and the orientation of the inlets and outlets at the base of the ventricles. The splined curve of valvar attachment is given a 0.8–1.2 mm thickness so that it is identifiable in the models.

For simulation of planned surgical procedure, the right ventricular free wall is kept intact except for a small hole in the apex for removal of the support material from the cavity and the large parts of the wall of the right atrium and left ventricle are removed (Figure 6). As discussed, cardiac valves are not able to be imaged adequately for 3D printing. For simulation purposes, fake valves are created using CAD tools (Figures 4 and 5). With the fake valves added to the model, the simulated surgical procedures may be felt more realistic.

The infant’s heart with DORV typically ranges between 5 and 10 cm along the long axis. For easier demonstration of the pathology, one might want to produce a magnified model of the whole or a part of the heart.

## Printing

The STL files are loaded to the software program of the 3D printer and the materials are assigned to the files for printing. For pure anatomical assessment of DORV, the models are printed with solid or, more favorably, flexible material. Among the existing 3D printers, the printers using PolyJet Technology and photopolymer resin materials (Objet Connex Series or J750 printer and TangoPlus FullCure resin, Stratasys Ltd., MN, USA, and ProJet 5500X printer and VisiJet CE NT-Elastomeric Natural resin, 3D Systems, Rock Hill, SC, USA) provide the physical properties of the printed models closest to those of human soft tissue, allowing simulated surgical and interventional procedures.

## Applications

**Figure 8:**
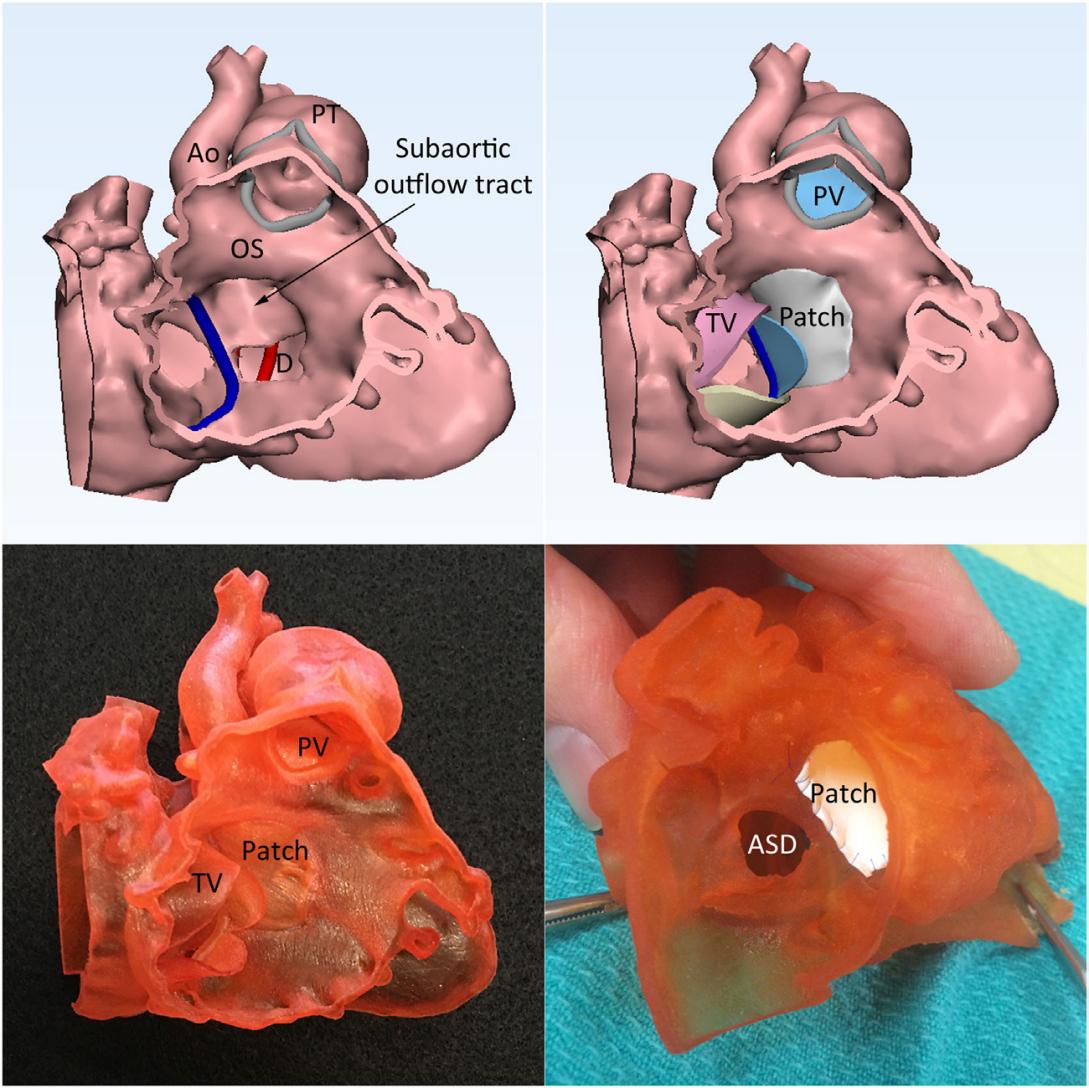
Double outlet right ventricle with a perimembranous ventricular septal defect (VSD) with predominantly inlet extension. Left upper panel shows that the spatial relationship of the VSD (D), outlet septum (OS), and the subaortic outflow tract. Right upper panel shows the graphically designed cardiac valve leaflets and intraventricular baffle patch. Left lower panel shows the 3D print model with the graphically designed patch added to the endocardial surface anatomy model of the right ventricle. Right lower panel shows the result of the application of surgical patch on the simulation model. Ao, aorta; ASD, atrial septal defect; PT, pulmonary trunk; PV, pulmonary valve; TV, tricuspid valve.

Echocardiography is the first line investigation tool that provides sufficient information for the general diagnosis and categorization of DORV. As the surgical anatomy of the cases with DORV is far from uniform, individualized approach to the surgical decision and procedure is more crucial than in any other forms of congenital heart disease. The major question to be answered is whether an unobstructed intraventricular baffle can be applied from the margin of the ventricular septal defect (VSD) to the aortic valve or, less frequently, to the pulmonary valve without compromising the right ventricular inlet dimension, the tricuspid valve (TV) function, both right and left ventricular outflow tracts, and the volume of the remaining right ventricle. Especially, when the VSD involves the inlet part of the right ventricle and/or the arterial valves are remote from the upper margin of the VSD due to long muscular infundibulum, imaging interpretation for feasibility of biventricular repair is not only challenging but also hard to explain (Figure 8). Furthermore, incomplete information or inappropriate interpretation may result in wrong choice in binary decision for biventricular versus univentricular repair. More than 50% of the cases referred to us for 3D printing were the patients with a primary diagnosis of DORV (3). Additional 15% cases had DORV in a more complex setting such as discordant atrioventricular connection and twisted heart. We find that 3D printing is not necessary in cases with classic subaortic or subpulmonary VSD. In other forms of DORV, there are a number of factors that should be considered for assessment of feasibility for biventricular repair (22). They include the following: (1) the exact location of the VSD within the septum, (2) the relationship of the VSD to the septal leaflet of the TV, (3) the commitment of the VSD to the subaortic or subpulmonary outflow tract, (4) the extent of the muscular infundibulum, (5) the distance between the upper margin of the VSD to the nearest arterial valve, and (6) the estimated volume of the remaining right ventricle after application of the intraventricular baffle. We often find that assessment of all these issues is difficult and can be imprecise. Non-uniform usage of the terms in description of the morphology in DORV further complicates communication among the imagers and surgeons. The more complex the anatomy, the larger the gap of understanding between the imagers and surgeons. In those situations, 3D print models eliminate most of the chances of misinterpretation and miscommunication and provide the opportunity for making the most precise decision (7–10).

The result of the intended surgery can be simulated by applying a graphically designed patch or baffle (Figure 8, right upper and left lower panels) (6, 23). In addition, the intended procedure can be performed on the patient-specific 3D print model before performing actual procedure on the patient (Figures 7 and 8, right lower panel).

3D print models are great resources for morphology teaching and hands-on surgical training (HOST) (6, 24). As the image data are from living patients, the collection of the cases cover almost full spectrum of malformation and any number of duplicated models can be reproduced. In the last 2.5 years, seven HOST courses have been organized at the society level or locally. In these meetings, 9 of 18 HOST models were DORV cases. The attendees of the courses invariably found that the models showed pathological features well and quality of the models were excellent or good for surgical simulation despite most felt that the physical properties of the models were different from those of human tissues (6). The attendees found the HOST course helpful in their skill development and thought that HOST should be included in the training program for congenital heart surgery.

## Future Direction

3D printing will be more widely applied in DORV patients with further improvement of imaging, postprocessing, and printing technology. The major limitation would be the high cost that is not covered by the government or insurance company. The cost will be gradually reduced with more wide spread utilization of the technique. With increased awareness of the availability of the technology, 3D printing is expected to be utilized whenever the surgical anatomy is not clear enough for surgeons’ decision. Eventually, the surgeons would not be comfortable in performing the procedure for complex DORV cases without seeing the models in advance and might want to practice the intended procedure on the models before the surgery. In addition, a surgical baffle can be designed and trimmed using 3D print models before the surgical procedure. 3D printing will gradually become a part of the standard care of DORV patients and covered by the government or insurance companies. HOST on 3D printing models is expected soon to be a part of congenital heart surgery fellowship program. DORV will be one of the most important congenital heart diseases that require HOST.

## Author Contributions

S-JY drafted the review article. GA reviewed and approved the content of article.

## Conflict of Interest Statement

The first author is the owner of a 3D printing company “3D HOPE (Human Organ Printing and Engineering) Medical” and the chief officer of the not-for-profit organization “IMIB-CHD (International Medical Image Bank for Congenital Heart Diseases).”
